# Repertory of the Back

**Published:** 1898-02

**Authors:** E. H. Wilsey

**Affiliations:** Parkersburg, W. Va.


### REPERTORY OF THE BACK.

E. H. Wilsey, M. D., Parkersburg, W. Va.

(Continued from page 35 )

**Menses,** pain, in back during the, Agar., *Calc-c.*, *Caust.*, *Croc.*, Kalm., *Hydrast.*, Phos., *Sars.*, Iod., *Bell.*, Coccul.. *Sulph.*, Thuja., *Nitric-ac.*, Helon.

— violent pain in small of back in morning, on rising from bed, so that she was unable to move during menses, *Lyco.*

— dragging pains in back before menses, worse sitting, better walking, with cutting and pain as if beaten and contracted, flow at first scanty, profuse and brown next day, with relief of pains, flow more profuse at night, Mag-c.

— pain in small of back as before menses with diarrhœa, Kali-iod.

— drawing pains in back with tense feeling as before menses, Mosch.

— violent headache during menses, Inula.

— dragging in back as before menses, followed by slight show, Canth.

**Mental,** weak feeling in small of back, worse from mental annoyances, *Calc-c.*

**Middle,** aching in middle of back with flashing of heat, Lappa.

**Morning,** pressive heaviness and weariness in back in morning, Petrol.
— dragging pain in back in morning on awaking, Myrica.
— bruised pain in small of back from morning till evening, better by going to bed, Nat-s.
— pains as if bruised in morning, *Droser.*
— severe pain in small of back in morning, Senecio.
— pain on motion in small of back, in bed night and morning, Croc.
— tearing pains in back, especially in morning, *Canth.*
— pain in back morning on rising, Ran-b.
— bruised pain in back from morning till 2 P. M., Mag-c.
— pain in small of back as from lameness in morning, Sel.
— violent pain in middle of back mornings on rising from bed, so that she was unable to move during menses, *Lyc.*
— stiffness and pain in upper dorsal and cervical muscles in morning, *Zinc.*
**Motion,** pain in back on motion, Amm-c.
— pain in small of back as if sprained on motion, *Puls.*
— a crick in back, better by continued motion, Sepia.
— spasmodic stitches in paroxysms in the middle of the back, which renders motion impossible for some minutes, Lyco.
**Move.** pain in small of back, cannot move, *Phos.*, Petrol.
— pain in back which does not allow him to move or stand, Petrol.
— sticking when sitting, with lightning-like pain above small of back, so that he cannot move in the evening, Nat-c.
**Moving,** drawing upward from foot, extending into back when moving, Nit-ac.
— excessive stiffness of one side of the neck extending to small of back, worse when moving, Guaic.
— a crick in back, worse when first moving, Sepia.
— produces intense pain in small of back, Kali-iod.

**Muscle**, a bruised sensation in muscles of loins and back, *Cham.*
— of back and face are principally affected, *Hydro-ac.*
— of both sides pain violently, *Casc.*
— stiffness and pain in cervical and upper dorsal muscles in morning, *Zinc.*

**Nausea,** pain in back, especially on bending backward or after a short walk, with nausea and weariness, Con.
— pain in back above hips with nausea and chills, Colocy.
— and tenesmus with backache, Mancin.
— feverish shiver every morning at 9, and some nausea, without subsequent heat, Mag-c.
— backache first low down, but passing off, he feels it higher up at 8 P. M., several times with nausea, Zing.
— backache causes nausea and faint feeling while standing, *Sep.*

**Neck,** pain from back going to neck, Lyss.

**Needles,** pricks as with fine needles from the back into the chest in the evening, Borax.
— excoriating, shooting on right half of the back as from needles, Platina.
— heat in the face transient with perspiration on forehead, and with heat on chest and in the back, combined with needle pricks, from within outward, most frequent and severe in the neck, Sarsa.
— stitches in back as from needles when sitting down, Caustic.
— severely itching rash on the nape, the back, and thighs always and more annoying after scratching, and pricking as from needles afterward, Mez.

**Neuralgia**, of back, *Cornus.*
— acute boring, darting, *Mag-p.*
— of back, running up more particularly on left side, *Nit-ac.*
— of back extends through to thoracic and abdominal walls, Sul.
— pain in back, particularly left side, *Nit-ac.*

**Neuralgia,** pains from below up, mostly left side of back and hip, *Eup-pur.*

**Nerve** in back seems to be paralyzed and numb, and during this numbing process it causes a good deal of pain very similar to pain from killing a nerve in a tooth, Calab.

**Night,** aching in back, especially at night, *Amm-m.*

— tearing in back from morning till night, Ant-cr.

**Nightmare,** while lying on his back, awaking with a scream, Guaic.

— pain especially at night in small of back, *Cham.*

— pain in back, most troublesome during night, *Helon.*

— pain in back and loins at night, Senecio.

— bruised pain at night in back, toward morning dare not turn over, Nat-c.

— pain in back at night, *Nat-m.*

— pain in back at night as if beaten to pieces, worse by motion, Mag-c.

— pain in back at night so he could not lie on small of back, Mag-c.

— pain in back and small of back at night as if broken, *Mag-c.*

— nightly pain in small of back and thighs, Mag-s.

— drawing pain in back, she had to turn over frequently to get better, Nat-m.

— pain in back, worse when lying down at night, Kreos.

— pain in lower part of back and head all night, Lact-ac.

— pain left side of back nights so she cannot lie upon it, Carbo-an.

— pain and stiffness in small of back at night, *Lyc.*

— tearing pain in back at night, Phos-ac.

**Noon,** a feeling at noon while walking as if a weight were lying across shoulders, weighing him down so that his head sunk forward, Carbon-s.

**Nocturnal** pains in small of back, which always awake her from sleep, *Amm-m.*

— relief of pains in back, hips, and thighs, Syph.

**Numbness**, severe headache all day, with numbness of hands, Lyss.
— in small of back, Berb.
— coldness and feeling of numbness on side of back on which he lays during his siesta, *Calc-c.*
— and weakness in back, *Agar.*
— pricking, causing a cold sensation in back, Ox-ac.
— sensation in small of back as if bruised or broken, Ox-ac.
— small of back and nates are very numb, Spong.
— in back with uneasiness, *Calc-phos.*
— in lower part of back and lumbago, Gnaph.
— of back, better by expulsion of wind, Berb.
— in back in intermittent fever, *Coccul.*
— nerve of back seems to be paralyzed and numb, and during the numbing process it becomes very painful, similar to killing a nerve in a tooth, Calab.
— pain in back of head and neck, with numbness under left scapula extending as a band down to left hip,Ailanth.
**Occiput**, pain in right side of back, with painful feeling in occiput, pressing aching toward left ear, *Chel.*
**Opisthotonus**, Canth., Oenan., *Opi.*, Stram.
**Oppression** and anxiety when she walks somewhat quickly, with perspiration on the back and chest, Nit-ac.
**Outstretched**, when lying still outstretched on his back he feels somewhat easier, Borax.
**Ovaries**, lancinating pain and weight in back, hips, and lower abdomen, extending from womb and ovaries, *Con.*
— weakness of back with ovarian pains, Abrot.
**Pain** in shoulder and back, *Hydras.*
— in small of back, *Kali-b.*
— in back, Lac-can.
**Palpitation**, with pain in small of back, *Lach.*
— violent while lying on the back, so that she woke up at 12 at night and sat up full of anguish, Nitrum.
— tabes dorsalis, especially in women, with great weakness of legs and back, *Graph.*

**Paralytic** drawing in spinous process of first dorsal vertebra, Staph.
— pain in small of back, better by lying on abdomen, worse bending backward, Selen.
— pain in small of back, Sabin.
— pain in small of back while sitting or standing, Coff-cr.
— pain in small of back with spasmodic drawing across hips, which prevents walking, with anxious, fearful mood, *Coccul.*
— pain in small of back as if beaten, *Nat-m.*
— pressing pain in small of back as if one had been lying in an uncomfortable position, worse on rising from a seat and beginning to walk, Zinc.
— or rheumatic lameness, stiffness after rest, better by gentle motion, *Kali-ph.*
— and weak sensation in small of back, Phos.

**Paralysis** in muscles of back up to neck, also of limbs, lower are œdematous but retain sensibility, *Cup.*
— weakness on early rising like paralysis in small of back and sometimes near abdomen, *Nat-m.*
— stiffness and paralysis in back and sacrum, Kali-c.

**Paralyzed,** nerves in back seem to be paralyzed and numb, and during this numbing process it causes a good deal of pain, very similar to the killing of a nerve of a tooth, Calabar.
— weakness in the back, and he feels paralyzed in lower limbs, Sil.
— sensation as if back were paralyzed, Physos.
— feeling in small of back extending to hips and left side, Zinc.

**Paroxysm,** pain in back before paroxysm, Bry.

**Pecking,** severe tearing or pecking pressure in the back with a chill, later passing over into a dull, pressive headache, with heat in head, *Sil.*

**Periodical,** pains in back, worse evening or warm room, better in cool or open air, Kali-s.

**Periodical** pains in back returning about midnight and extending to the head, *Chin-sul.*

— unbearable pains in back, recurring periodically and hindering walking, Phos.

**Perspired,** sensation of heat in the whole body, especially in back, where she imagined she perspired, Zinc.

— very profusely on his back when he walks or exercises otherwise, Nat-c.

**Perspiration,** profuse at night on back, Guaj.

— nocturnal emission of semen and perspiration on back with waking up at about 2 A. M., Sil.

— heat in the face transient with perspiration on the forehead, with heat on chest and on back combined with needle pricks, Sars.

— sudden heat on the whole body while sitting down, and soon after followed by perspiration, with pupils very much contracted, Mang.

— and some itching on the back, Phos-ac., Caust.

— on neck and back from least exertion, *China.*

— oppression and anxiety when she walks somewhat quickly, with perspiration on back and chest, Nit-ac.

**Pinching** now in dorsal and now in abdominal muscles, Peonia.

— together in the fleshy part of the back both at rest and in motion, Nit-ac.

— in middle of back, Cann-s.

— and cutting below the navel with shivers over the back, then heat in the head and urging to stool at noon, Mag-m.

— and shooting on false ribs in back, Stann.

— and pressure in right side of back, Lyco.

— pains in right side of back for an hour, Sil.

— and burning pains in small spots of back, Zinc.

— pains in the back in the evening, Nitrum.

**Pinching** and shooting pains at night, now below the chest, and now in back, Nit-a.

— in abdomen as if sore morning in bed, then pressive pain as if sore in back and scapula, ceasing after rising, Nat-m.

— in stomach, which contracts the chest, with grasping together of the back, awaking her in morning from sleep, Con.

— pain middle of back as if some one was pinching it with pincers, pain extending gradually toward abdomen, Can-s.

**Pinches,** several sharp smarting pinches on the back part of the ribs on both sides of the back, Kali-c.

**Pieces**, pain in small of back as if broken to pieces, in morning, in bed, Staph.

— severe pain in back at night in bed as if it were beaten to pieces, worse when moving, but feels it when at rest, Mag-c.

**Pimples** and itching on small of back, Niccol.

— eruption of pimples on back with itching in evening in bed, Nat-m.

**Plug,** severe pain in small of back, she was unable to sit, then sense of a plug in back, had to put a pillow under it, *Carbo-v.*

**Pollutions,** at night he always lies upon his back, tossing about with frequent awaking and frequent pollutions, Dig.

**Position**, restless sleep, cannot lie in any position, only on back, Dig.

— pain in back as if he had lain in an uncomfortable position. *Puls.*

— acute pain in back gradually extending down into thighs, with great torture, seeks relief by change of position, Ox-ac.

**Pregnancy,** pain in back and lower ribs during pregnancy, *Arg-n.*

**Pressing** pain in small of back, *Spong.*

— pain in back comes up between shoulders, is felt as a throbbing, Tereb.

— constant pain in small of back, worse by motion, Psor.

— cramp-like pain in small of back in region of kidneys, *Caustic.*

— severe in back and down thighs, and through hips with heavy pressing down, *Cimic.*

— spasm in back with severe pressing and drawing, Con.

— from above downward, a pain in left side of back above hip, Stann.

— tearing and gnawing in back, while walking, after rising from a seat, Canth.

— aching in back at lower border of scapula, Rumex-crisp.

— running from between shoulders to occiput and across right ear in forenoon, Lyss.

— a feeling in small of back in morning as if pressed inward, *Kali-c.*

— dull headache in afternoon, as from sitting too long, Pallad.

— collection of wind pressing against back, Cup-sul.

**Pressive** pain in back between scapula, as if the parts had been strained or had suffered injury, with a like pain in front part of chest on moving arm, Carbo-a.

— tearing pain in left side, beside the hip, extending into the back, Carbo-v.

— pain in middle of back between scapula, Calc-c.

— he awakes at 2 A. M. with a febrile rigor and hot dry skin, from time to time a shivering ague from the nape down the back and over the chest; then some sleep, from which he awakens in a gentle perspiration, with a pressive pain in back, as also in and behind the hips and in the abdomen, with inclination to vomit, Hepar.

**Pressive** and pinching pains in right side of back, Lyco.
— pain beside the lowest part of the back, Carbo-v.
— pain as from a bruise on the lower part of back, with severe pressure in the scrobiculus cordis, same in motion and at rest, Colocy.
— pain in small of back, *Carbo-an.*
— a severe pain in back under the right scapula, which at inspiration changes into a lancinating pain, Cup.
**Pressure,** hard in small of back, *Kali-c.*
— in small of back below scapula one inch from spine, Lyss.
— worse backache, Verbasc.
— tired, bruised feeling in muscles of back, extending from points of scapula to ilium on each side of spine, better by firm pressure, Vib-op.
— in back, Amm-c.
— in back between scapula, Graph.
— burning in small of back at 10 P. M., Cepa.
— heaviness, worse by sitting, better by motion and pressure, *Aloe.*
— in small of back, Variol., Clem.
— tensive upon a small spot on back by border of right scapula, Zinc.
— strong before the protrusion of the varix from the rectum, Alum.
— slight on last dorsal vertebra increases pain exceedingly, *Arm-sat.*
— pain in muscles of back, a pressure, Euphorb.
— pain beside spine with pressure, Graph.
— pain back augmentary with violent pressure on stooping down, better by rest, but breaking out again with every turn of the body, Sarsa.
— the left side of the back is painful, as from pressure on an inflamed spot, Nat-m.
— inward in back and chest up to neck, muscles and vessels seem contracted, Opium.

**Pressure** and heaviness in small of back as if one had received a blow, worse while sitting, *Rhus-t.*

— as with a cutting edge across small of back while standing or bending backward, Rhus-t.

— in middle on left side of back, as from much stooping, Mur-ac.

— in back opposite bowels, then oppressive stitches on least motion or breathing, so that he had to walk bent, when lying still griping as in a malignant ulcer and with oppression of chest, Arg-m.

— drawing pressure in back, Kali-c.

— sharp in upper part of back, Kali-c.

— in small of back, with leaden-like heaviness of lower limbs, *Camph.*

— in shoulder and on back, Petrol.

— in middle of back, Caustic.

— and burning in back, better by walking, worse by sitting and by lying in bed, Nitrum.

— frequent extending to throat and back, Mag-m.

— hard in left dorsal muscles near spine, Staph.

— severe backache, better from pressure and lying on it, *Nat-m.*

— dull and slowly intermittent dull thrusts in the middle and left side of the back, Platina.

— at night in the intensely inflated abdomen, worse by any other than position on back with compressed breath and quickened pulse, Mez.

— great pressure in back from within outward during menses, *Nux-m.*

**Pricks** as with fine needles from the back into chest in evening, Borax.

**Pricking** and numbness, causing a cold sensation in back, Ox-ac.

— in skin of back, Sil., Aur-mur.

**Prickly,** burning and heat like prickly heat on back, Apis.

**Ptyalism,** pain in small of back with ptyalism, Cinnam.

**Pulling** and tearing in back and legs, *Ars.*

**Pulsating,** and cutting feeling in back, *Nat-m.*

— and throbbing in back, *Lyco.*, Bar-c., Thuya.

**Pulsation** or cramp-like contraction ascending from the thighs into the small of back, Ruta.

— in middle of back, Caustic.

— in small of back, *Sepia.*

**Pustule** on small of back, Nat-c., Calc-c.

**Quivering,** a thrilling quivering about an inch to the left of the dorsal vertebra below scapula in evening, on rising from reclining it changed into aching, later crawling with a thrilling, Ver-v.

**Rash,** severely itching on the nape, the back, and thighs, always worse and more gnawing after scratching and pricking as from needles afterward, Mez.

**Rattling,** slight in chest at night when lying on back, Kali-c.

**Rectum,** soreness in small of back and rectum with diarrhœa, Jatropha.

**Red,** spots like ringworm on back, All-sat.

— pain in back as if a red-hot iron was thrust through lowest vertebra, Alum.

**Rest,** during rest bruised pain in back, Kali-c.

— small of back pains, worse lying on back, better by rest, Lappa.

— stitches and pain in small of back as from sprain during rest and ceasing on walking, Staph.

— pain in small of back during rest, Alum.

— drawing pain from small of back down thighs during rest; stitches when moving, better by pressure, *Dulc.*

**Restlessness** with coldness in back at night, Nat-m.

**Respiration** difficult, especially when lying on back in goitre, *Iodum.*

— burrowing in the dorsal muscles during respiration, Stann.

**Respiration,** dull stitch in back near scapula (right) impeding respiration, most perceptible when moving, Mez.

— single violent stitches in the upper part of the back in respiring, Calc-c.

**Rheumatism** of back, *Actea-r.*, Bell., *Bry.*, Chrom-c., *Coloc.*, *Cornus.*, *Hep.*, *Nux-v.*, *Rhod.*, *Rhus-t.*, *Phyto.*, *Ars.*, Pet., *Ruta.*, *Dulc.*, *Rhus-v.*, Sang.

— of back during climacteric period, Ustil.

— of back and shoulder, Ustil.

— of dorsal muscles, Ruta.

— chronic rheumatism of back and chest, Kali-iod.

— in small of back for several weeks, *Phyto.*

— of back, better after heat, Aur-met.

— of back, worse toward morning, *Nux-v.*, Petrol., *Ruta.*

— worse on motion, Variol.

— wandering rheumatism of back, Lyc-vir.

— stiffness in small of back, pains like rheumatism, Iod.

— pain in muscles of back like rheumatism, Variol.

**Rheumatic** drawing in back, especially when stooping for several days, Carbo-v.

— tension in back and in right side of chest, Lyco.

— drawing from upper dorsal vertebra to left clavicle in afternoon, with pain on touch, Euphras.

— pains in bones and joints of back and limbs at night, Gels.

— pains and stitches in back leave a burning, Comocladia.

— pains in back while stooping, felt as if something cracked across sacrum, cannot stoop or move for pain, which remains constant even while at rest, but on least movement of trunk or legs, *Kali-b.*

— pains and stitches in back, *Hepar.*

— pains in neck and back, *Colchi.*

— pains in small of back and heart region, *Cact.*

— drawing pains in back, worse when stooping, *Carbo-v.*

— stiffness in whole left side of back from the nape down into the sacrum, with unbearable pain at the least motion in turning of parts, Guaj.

**Rheumatic** pains about right shoulder and various parts of back, Chrom-ac.

— pains in back and shoulders, *Rhod.*

— pains in back followed by burning, Bapt.

— pain in back, Bell.

— pains in side of back, Ambros.

**Riding,** aching when riding in a carriage, *Nux-v.*

— a jar from riding or a misstep hurts her back severely, Sepia.

**Rigid,** muscles of back rigid, even a tetanic condition may ensue, Physos.

**Rigidity,** in the back, Petrol., Stram.

**Rise,** scarcely able to rise or stoop after sitting, *Æscul.*

— pain in small of back, after sitting can scarcely rise, *Puls.*

**Rising,** after rising the whole back was painful so that she could scarcely move, with weariness of limbs, aversion to eating and working, with shivering chilliness without thirst, Hepar.

— soreness in small of back on rising, with rumbling in abdomen, Fer.

— in small of back when rising, after having been sitting; a violent pain which hinders his rising, also impedes the motion of the thighs, Agari.

— violent pain in small of back while rising from sitting, disappeared when walking, Ant-cr.

— cracking sound from first cervical vertebra on rising from sitting, with stiffness in left side of neck, Zing.

— stiffness in back on rising in morning, Sul-ac.

**Ringworm,** red spots like ringworm on back, All-sat.

**Rolling,** cutting in small strip in side of abdomen over against the back, then rolling in abdomen while the pain goes off, Sarsa.

**Rubbing,** burning small spot in small of back, better from rubbing, *Phos.*

**Rumbling,** itching, crawling and smarting in whole of back, followed by rumbling, Alum.

**Scream,** nightmare while lying on back awakens him with a scream, Guaj.

— violent pain in small of back when making least bodily effort, sometimes obliging him to scream, *Calc-phos.*

**Screwed,** pain in small of back as if screwed in, *Kali-iod.*

— in sensation on rising from a seat, in small of back, Zinc.

**Seat,** pain in small of back on rising from a seat, Sul.

— pain in small of back, ceases when rising from a seat, Cyclam.

— stiffness from pain in small of back when rising from seat, *Sil.*

— pain in small of back as after overlifting, worse at rest, night and in morning and when rising from a seat, *Staph.*

— insupportable backache toward evening and on rising from a seat, *Ars.*

**Seminal,** backache with seminal emissions, *Dig.*, *Kali-brom.*, *Kob.*, *Sarsa.*

— backache feels better after an emission, Zinc.

**Sensitiveness,** and pain in occiput, upper part of back and upper chest after a severe jarring, *Glon.*

— of upper part of back and neck, to touch or from lying on it, with swelling and heat, *Glon.*

— sweat on back with sensitiveness to cold air in morning, Chim-m.

— as if paralyzed in back and neck, *Coccul.*

— during short intervals of consciousness complains of great sensitiveness of back and neck and whole spine, *Coccul.*

**Severe,** puts right arm to small of back and draws mouth as if he had severe pain, Stram.

**Sewing,** pain in back, worse when sewing, Secale.

**Sexual,** backache from sexual excess, *Calc-c.*, *China*, *Nat-m.*, *Nux-v.*, *Phos.*, *Phos-acid.*, *Puls.*, *Sep.*, *Staph.*. Carbo-v., Sul.

**Sharp** pain in back, Merc-i-fl., *Con.*

— pain in back with chills in dysmenorrhœa, *Con.*

— neuralgic pains in back, Iod.

— pain shooting through back, *Lith.*

— pain in back running up, with constriction of anus, *Coloc.*

— pains in back rendering motion when not in an erect position intolerable, Tereb.

— spasmodic pain in back running to crest of left ilium, *Helon.*

**Shifting,** pains in back, worse when drawing a long breath, Sang.

— pains in back, intercostal neuralgia, *Mag-ph.*

